# Convergent Immune–Coagulation Programs Underlie Gastrointestinal Bleeding Risk in Portal Vein Tumor Thrombosis–Associated Hepatocellular Carcinoma and Portal Hypertension

**DOI:** 10.1155/humu/7210691

**Published:** 2026-05-12

**Authors:** Xu Rutao, Xiong Zhuxiang, Zhao Jichun, Zheng Tinghui, Zhang Kewei, Wang Qingqing, Yuan Ding, Chen Xiyang, Weng Chengxin, Huang Bin

**Affiliations:** ^1^ Department of Vascular Sugery, West China Hospital, Sichuan University, Chengdu, Sichuan, China, scu.edu.cn; ^2^ Department of Vascular Surgery, Henan Provincial People′s Hospital, Zhengzhou, Henan, China, zzu.edu.cn; ^3^ Department of Cardiac and Macrovascular Surgery, Nanchong Hospital of Beijing Anzhen Hospital, Capital Medical University, Nanchong, China, ccmu.edu.cn; ^4^ Department of Mechanics and Engineering, College of Architecture & Environment, Sichuan University, Chengdu, Sichuan, China, scu.edu.cn

**Keywords:** gastrointestinal bleeding risk, hepatocellular carcinoma, immune–coagulation dysregulation, portal hypertension, portal vein tumor thrombosis

## Abstract

**Background:**

Portal vein tumor thrombosis (PVTT) and portal hypertension are major contributors to gastrointestinal bleeding in hepatocellular carcinoma (HCC), yet the molecular programs linking vascular pathology, immune dysregulation, and bleeding risk remain incompletely defined.

**Methods:**

Bulk transcriptomic datasets related to PVTT (GSE77509 and GSE69164) and noncirrhotic portal hypertension (GSE77627) were analyzed using differential expression and weighted gene coexpression network analysis to identify robust disease‐associated genes. A six‐gene core signature was derived by intersecting differentially expressed genes with intramodular hub genes across datasets. Bleeding risk–associated biological programs, including coagulation, complement activation, angiogenesis, hypoxia, and inflammatory signaling, were quantified using ssGSEA. Immune infiltration was estimated using CIBERSORTx. Single‐cell RNA‐sequencing data from PVTT (GSE149614) were analyzed to resolve cell type–specific expression patterns and intercellular communication. Associations with survival, DNA methylation, immune infiltration, drug sensitivity, and molecular interactions were evaluated using public cancer genomics resources. Functional validation was performed using siRNA‐mediated knockdown and drug treatment assays in HepG2 and Huh7 cells, followed by proliferation, colony formation, and wound healing assays.

**Results:**

Bleeding risk–related biological programs exhibited dataset‐specific activation patterns and correlated with expression of the six‐gene signature. Single‐cell analysis revealed heterogeneous, cell type–specific expression across malignant, stromal, endothelial, and immune populations. OGFRL1 and WDR62 were significantly associated with overall survival and showed methylation‐linked transcriptional regulation. Genetic silencing or pharmacological targeting of these genes significantly suppressed HCC cell proliferation, clonogenicity, and migration in vitro. Drug signature analysis and molecular docking supported potential interactions of nocodazole with OGFRL1 and testosterone with WDR62, which phenocopied knockdown effects.

**Conclusion:**

These findings identify immune–coagulation dysregulation as a molecular link between PVTT, portal hypertension, and gastrointestinal bleeding risk in HCC and functionally validate OGFRL1 and WDR62 as biologically and therapeutically relevant targets.

## 1. Introduction

Hepatocellular carcinoma (HCC) is among the leading causes of cancer‐related mortality worldwide, largely due to its aggressive biological behavior and frequent association with vascular complications [1]. One of the most devastating manifestations of advanced HCC is portal vein tumor thrombosis (PVTT), which is characterized by direct invasion of tumor cells into the portal venous system [2]. PVTT markedly worsens prognosis by accelerating intrahepatic dissemination and by promoting portal hypertension [3]. Clinically, patients with PVTT frequently develop portal hypertension–related sequelae, including splenomegaly, ascites, and gastroesophageal varices. These conditions substantially increase the risk of life‐threatening gastrointestinal bleeding [4]. Despite its clear clinical relevance, the molecular mechanisms linking PVTT, portal hypertension, immune dysregulation, and bleeding susceptibility remain incompletely understood.

Portal hypertension is no longer viewed as a purely hemodynamic consequence of increased intrahepatic resistance but rather as a complex, multifactorial process driven by inflammation, endothelial dysfunction, aberrant angiogenesis, coagulation imbalance, and immune cell remodeling within the liver microenvironment [5]. In the context of HCC with PVTT, tumor‐driven vascular remodeling and inflammatory signaling further exacerbate portal venous pressure and destabilize the vascular wall, predisposing patients to hemorrhagic complications [6]. Emerging evidence suggests that immune–coagulation crosstalk, hypoxia‐inducible pathways, and extracellular matrix remodeling collectively contribute to variceal fragility and bleeding risk. However, most studies have examined these processes without integrating tumor‐intrinsic transcriptional programs, stromal interactions, and immune infiltration patterns across disease states. High‐throughput transcriptomic profiling has enabled systematic interrogation of gene expression alterations associated with HCC progression and vascular invasion [7]. Differential expression (DEG) analyses have identified numerous oncogenic drivers and stromal markers associated with PVTT. But still, reproducibility across independent cohorts remains a persistent challenge. Network‐based approaches, such as weighted gene coexpression network analysis (WGCNA), provide a complementary framework by identifying gene modules that reflect coordinated biological programs [8]. These approaches are particularly valuable in heterogeneous diseases such as HCC, where complex interactions between tumor cells, endothelial compartments, and immune populations shape disease trajectory. Integrating differential expression with coexpression networks across multiple datasets offers a robust strategy to identify core molecular signatures that are consistently associated with PVTT and portal hypertension–related pathology.

Beyond bulk transcriptomics, single‐cell RNA sequencing has transformed the understanding of cellular heterogeneity within the tumor microenvironment. scRNA‐seq studies in HCC have revealed extensive diversity among malignant hepatocytes, endothelial cells, fibroblasts, and infiltrating immune populations. These contribute distinct signaling cues that regulate angiogenesis, inflammation, and immune evasion [9]. Importantly, cell–cell communication analyses have highlighted ligand–receptor interactions that drive vascular remodeling and immune suppression in advanced disease [10]. However, the specific cellular contexts in which PVTT‐associated genes operate and how these genes participate in intercellular signaling networks relevant to portal hypertension and bleeding risk remain insufficiently characterized. Immune infiltration represents another critical axis linking PVTT, tumor progression, and vascular complications. Alterations in the balance of cytotoxic lymphocytes, macrophage subsets, and regulatory immune cells have been associated with both tumor aggressiveness and fibrosis‐driven portal hypertension [11]. Computational deconvolution methods applied to bulk transcriptomic data allow estimation of immune cell proportions and facilitate correlation analyses between gene expression and immune landscapes [12]. Coupled with epigenetic analyses, including DNA methylation profiling, these approaches can uncover regulatory mechanisms that modulate immune–tumor interactions and influence clinical outcomes. From a translational perspective, identifying molecular determinants that not only stratify prognosis but also suggest therapeutic vulnerabilities is of high priority. Integrative analyses combining survival data, somatic mutation profiling, drug sensitivity correlations, and protein–protein interaction networks can refine candidate targets and inform drug repurposing strategies [13]. Structure‐based molecular docking further provides mechanistic support by evaluating the physical feasibility of protein–ligand interactions for prioritized gene–drug pairs.

In this study, we employed a comprehensive, multiscale analytical strategy to dissect the molecular landscape linking PVTT, portal hypertension, immune dysregulation, and bleeding risk–associated biological programs. By integrating differential expression analysis, WGCNA‐based network modeling, pathway‐level quantification of coagulation and inflammatory programs, single‐cell transcriptomic profiling, cell–cell communication inference, immune infiltration estimation, epigenetic and survival analyses, and structure‐guided drug interrogation, we systematically identified and prioritized a small set of genes with consistent relevance across datasets and disease contexts.

## 2. Methodology

### 2.1. Differential Gene Expression Analysis

Publicly available transcriptomic datasets related to PVTT (GSE77509 [14] and GSE69164 [15]) and HTN (GSE77627) were analyzed using the limma package in R [16]. Expression matrices were log2‐transformed, and linear models were fitted for each gene using group‐specific design matrices. DEGs were defined using the Benjamini–Hochberg false discovery rate (FDR) < 0.05 and an absolute log2 fold change (|log2*F* *C*|) > 1 [17]. Transformation and integration were performed at the level of statistically derived features to minimize batch‐effect–driven confounding. Genes consistently upregulated or downregulated across datasets were selected for downstream analysis.

### 2.2. WGCNA

WGCNA was performed independently for each dataset using the WGCNA R package [18]. Candidate powers ranging from 1 to 20 were evaluated, and the selected soft‐thresholding powers (*β*) were six for GSE77509, five for GSE77627, and seven for GSE69164, determined based on the scale‐free topology criterion. The adjacency matrix was converted into a topological overlap matrix (TOM), and hierarchical clustering was applied to the TOM‐based dissimilarity matrix [19]. Module identification was carried out with a minimum module size of 30 genes, a signed TOM, and dynamic tree cutting. Closely related modules were merged using a module eigengene dissimilarity threshold of 0.25 to reduce redundancy. Module eigengenes were calculated and ordered to summarize the expression behavior of each module [20]. Module–trait relationships were assessed by correlating module eigengenes with binary clinical traits using Pearson′s correlation. *p* values were computed based on Student′s *t*‐distribution [21]. Hub genes within each module were identified based on module membership (kME) calculated as the correlation between individual gene expression profiles and their corresponding module eigengenes. For each module, genes were ranked by the absolute value of kME, and the top‐ranking genes were designated as intramodular hub genes.

Hub gene lists derived from WGCNA and differentially expressed genes were intersected across all three datasets. Only genes consistently detected DEG significance criteria (FDR < 0.05 and (|log2*F* *C*| > 1) with concordant direction of change and were also identified as intramodular hub genes (high kME) within their respective WGCNA modules across the two PVTT datasets and the noncirrhotic portal hypertension dataset were retained. This yielded a six‐gene core signature consisting of RBM27, WDR62, OGFRL1, STRN, GPX1, and FBXO38.

### 2.3. Quantification of Bleeding Risk Proxy Biological Programs

To explicitly quantify molecular programs implicated in gastrointestinal bleeding risk, single‐sample gene set enrichment analysis (ssGSEA) was performed using the GSVA framework [22, 23]. Hallmark gene sets representing coagulation, complement activation, inflammatory response, IL6–JAK–STAT3 signaling, TNF*α* signaling via NF‐*κ*B, hypoxia, and angiogenesis were obtained from MsigDB [24]. This allowed us to get continuous pathway‐level activity estimates that reflect upstream biological processes known to drive portal hypertension, variceal fragility, and hemorrhagic risk.

## 3. scRNA‐Sequencing Analysis

Publicly available scRNA‐seq data were obtained under Accession Number GSE149614 [25]. Only cells derived from tumors and tumor‐associated samples were retained for downstream analyses. Raw count matrices were imported into Seurat, and cells with fewer than 200 detected genes or greater than 10% mitochondrial gene content were excluded from downstream analysis. Cellular neighborhoods were constructed using a shared nearest‐neighbor graph, followed by graph‐based clustering to identify transcriptionally distinct cell populations. Uniform Manifold Approximation and Projection (UMAP) was applied to visualize cellular relationships in low‐dimensional space [26]. Our six genes were evaluated across annotated cell types. Pseudobulk expression profiles were computed using average expression per cell type, followed by row‐wise scaling [27].

### 3.1. Cell–Cell Communication Inference

CellChatDB.human was used as the ligand–receptor interaction reference [28]. Overexpressed genes and interactions were identified within each cell group prior to communication probability estimation. Cell–cell communication probabilities were computed using a truncated mean approach, followed by pathway‐level aggregation of ligand–receptor interactions. Network centrality measures were computed to quantify the relative signaling roles of each cell type as signal senders, receivers, mediators, or influencers.

## 4. Immune Infiltration Analysis

To estimate immune cell infiltration, CIBERSORTx was applied using the LM22 leukocyte gene signature matrix, which profiles 22 distinct human immune cell subsets [29]. CIBERSORTx outputs were visualized in R, and gene expression values for our six genes were aligned with CIBERSORTx immune fraction estimates. Spearman′s rank correlation analysis was performed between the expression level of each selected gene and the estimated fraction of each of the 22 immune cell types [30]. These correlation analyses are descriptive and do not imply direct regulatory relationships, as observed associations may reflect indirect effects or shared upstream transcriptional programs.

### 4.1. Expression Profiling Across Pathological States in LIHC

The mRNA expression patterns of the six shortlisted genes were evaluated using the GSCA platform [31]. Gene expression levels were also examined across pathological states relevant to LIHC to characterize disease‐associated transcriptional alterations.

## 5. Immune Associations, Methylation, and Drug Sensitivity Analysis

Using GSCA, correlations between gene expression levels and immune cell infiltration in LIHC were computed to assess immune–tumor associations. Correlations between DNA methylation levels of each gene and immune infiltrates were analyzed to explore potential epigenetic regulation of immune interactions. Drug response analysis was performed by correlating gene mRNA expression with CTRP drug sensitivity scores [32].

### 5.1. Survival Analysis and Prioritization of Prognostic Genes

Overall survival analyses were conducted for the six candidate genes in LIHC using the KM plotter tool [33]. Only two genes, OGFRL1 and WDR62, had an FDR below 5% and were prioritized for subsequent analyses.

### 5.2. Somatic Mutation, Methylation, and Expression Correlation Analyses in Liver Cancer

Somatic mutation frequencies of OGFRL1 and WDR62 were assessed using the GSCA database. In addition, overall survival analyses based on gene methylation status were performed in liver cancer cohorts. Correlations between gene methylation levels and corresponding mRNA expression were also evaluated to determine whether epigenetic regulation contributed to transcriptional dysregulation of these genes in liver cancer.

### 5.3. PPI Network Construction and Functional Enrichment Analysis

Protein–protein interaction networks for OGFRL1 and WDR62 were constructed using the STRING database [34]. Functional enrichment analyses were subsequently analyzed to identify significantly associated Gene Ontology biological process and cellular component terms.

### 5.4. Drug Signature Identification

Drug–gene associations were identified using the Enrich Drug Signature Database [35], from which two candidate compounds were prioritized. Testosterone was identified as a potential interacting compound for WDR62, while nocodazole was identified for OGFRL1. Chemical structures of both compounds were retrieved from PubChem [36], with PubChem CIDs 6013 for testosterone and 4122 for nocodazole. Protein structures for WDR62 and OGFRL1 were obtained from AlphaFold using the AlphaFold identifiers AF‐0433792‐F1‐V6 for WDR62 and AF‐Q5DC844F1S‐V6 for OGFRL1 [37].

## 6. Molecular Docking

Molecular docking analyses were performed to evaluate the binding feasibility and structural compatibility between each target protein and its corresponding small‐molecule compound using CB‐Dock2 [38]. Blind docking was conducted to identify potential binding cavities, followed by docking score estimation and pose ranking based on predicted binding affinity. The top‐ranked docking poses were retained for the interpretation of protein–ligand interactions.

## 7. Cell Culture and Maintenance

Human HCC cell lines HepG2 and Huh7 were used for in vitro functional experiments. HepG2 cells were obtained from the American Type Culture Collection (ATCC, HB‐8065), while Huh7 cells were obtained from the Japanese Collection of Research Bioresources (JCRB0403). Cells were cultured in high‐glucose Dulbecco′s Modified Eagle Medium (DMEM; Gibco, Thermo Fisher Scientific, Cat# 11965092) supplemented with 10% fetal bovine serum (FBS; Gibco, Thermo Fisher Scientific, Cat# 26140079) and maintained at 37°C in a humidified incubator with 5% CO_2_. Cells were routinely passaged at 70%–80% confluence and used at low to mid passages for all experiments. To ensure cell culture quality, all cell lines were routinely tested for mycoplasma contamination using the MycoSEQ Mycoplasma Detection Kit (Applied Biosystems, Thermo Fisher Scientific, Cat# 4460623) according to the manufacturer′s instructions, and only mycoplasma‐negative cells were used for subsequent assays.

## 8. Drug Treatment

To functionally evaluate candidate compounds identified through drug signature analysis and molecular docking, nocodazole and testosterone were selected as representative small molecules associated with OGFRL1 and WDR62, respectively. Nocodazole, a microtubule‐disrupting agent identified as a top‐ranked compound associated with OGFRL1 expression profiles, was purchased from Sigma‐Aldrich (Merck) (e.g., nocodazole, ≥ 98% purity, Cat# M1404). A 10 mM stock solution was prepared in dimethyl sulfoxide (DMSO) and stored at −20°C. For cell treatment experiments, nocodazole was used at a final concentration of 100 nM, a dose widely reported in the literature to modulate microtubule dynamics and cellular behavior in HCC and other solid tumor cell lines without inducing excessive cytotoxicity. Testosterone, identified through drug signature enrichment and molecular docking as a potential interacting compound for WDR62, was obtained from Sigma‐Aldrich (Merck) (e.g., testosterone, ≥ 98% purity, Cat# T1500). Testosterone stock solutions (10 mM) were prepared in DMSO and stored at −20°C in accordance with the manufacturer′s instructions. Cells were treated with testosterone at a final concentration of 10 nM, a physiologically relevant dose commonly used to assess steroid hormone–mediated effects on proliferation and migration in HCC models. For all experiments, HepG2 and Huh7 cells were seeded and allowed to adhere overnight prior to drug exposure. Control cells received an equivalent volume of DMSO, with the final DMSO concentration maintained below 0.1% across all conditions.

## 9. Gene Knockdown Experiments

For transient gene knockdown, small interfering RNAs (siRNAs) targeting WDR62 and OGFRL1 were transfected using Lipofectamine RNAiMAX Transfection Reagent (Invitrogen, Thermo Fisher Scientific, Cat# 13778150) following the manufacturer′s protocol. Briefly, cells were seeded 24 h prior to transfection to achieve approximately 60%–80% confluence. siRNAs were used at a final concentration of 10 nM, and a nontargeting siRNA was included as a negative control. At least two independent siRNA sequences per gene were used to minimize off‐target effects.

### 9.1. RNA Extraction and RT–qPCR

Total RNA was extracted using TRIzol Reagent (Invitrogen, Thermo Fisher Scientific) according to the manufacturer′s protocol. RNA concentration and purity were assessed spectrophotometrically, and equal amounts of RNA were reverse transcribed using the High‐Capacity cDNA Reverse Transcription Kit (Applied Biosystems, Thermo Fisher Scientific, Cat# 4368814). Quantitative real‐time PCR was performed using TaqMan Fast Advanced Master Mix (Applied Biosystems, Thermo Fisher Scientific) on a real‐time PCR system. Gene expression was measured using the following TaqMan Gene Expression Assays: OGFRL1 (Hs00226193_m1), WDR62 (Hs01063714_m1), and GAPDH (Hs02786624_g1) as an endogenous control. Relative gene expression levels were calculated using the 2^−*Δ* *Δ*Ct^ method and normalized to GAPDH. All experiments were performed with at least three biological replicates and technical triplicates. GAPDH was selected based on its stable expression across siRNA‐mediated knockdown and drug treatment conditions, as confirmed by minimal variation in Ct values across experimental groups.

## 10. Western Blot Analysis

Total protein was extracted using RIPA lysis buffer (Thermo Scientific/Pierce) supplemented with protease inhibitors, and protein concentrations were determined using the Pierce BCA Protein Assay Kit (Thermo Scientific/Pierce). Equal amounts of protein were separated by SDS–PAGE and transferred onto PVDF membranes. Membranes were blocked and incubated overnight at 4°C with primary antibodies against WDR62 (PA5‐31552, Thermo Fisher Scientific) or OGFRL1 (89‐208‐825, Thermo Fisher Scientific). GAPDH (MA5‐15738) was used as a loading control. After incubation with HRP‐conjugated secondary antibodies (Thermo Fisher Scientific), protein bands were visualized using Pierce ECL Western Blotting Substrate and quantified by densitometry using ImageJ software. Protein expression levels were normalized to the corresponding loading controls.

### 10.1. Cell Proliferation Assay

Cell proliferation was assessed using the CyQUANT Direct Cell Proliferation Assay (Invitrogen, Thermo Fisher Scientific, Cat# C35011). Transfected cells were seeded into 96‐well plates at equal densities, and fluorescence intensity was measured at 0, 24, 48, 72, and 96 h according to the manufacturer′s instructions. Proliferation rates were calculated relative to control groups.

### 10.2. Colony Formation Assay

For colony formation assays, transfected cells were seeded into six‐well plates at low density (500–1500 cells per well) and cultured for 10–14 days with medium replaced every 2–3 days. Colonies were fixed with methanol and stained using crystal violet (Fisher Chemical, Thermo Fisher Scientific). Colonies containing more than 50 cells were counted, and colony numbers were quantified using ImageJ.

### 10.3. Wound Healing Assay

Cell migration was evaluated using a wound healing assay. Transfected cells were grown to near confluence in six‐well plates, and a linear scratch was created using a sterile pipette tip. Cells were gently washed with PBS to remove debris and incubated in serum‐reduced medium. Images of the wound area were captured at 0 and 24–48 h using an inverted microscope. Wound closure was quantified as the percentage reduction in wound area relative to baseline. To minimize the contribution of cell proliferation to wound closure, assays were performed under serum‐reduced conditions.

## 11. Statistical Analysis

All experiments were performed with at least three independent biological replicates. Data are presented as mean ± standard deviation. Statistical analyses were conducted using Student′s *t*‐test for two‐group comparisons or one‐way ANOVA for multiple comparisons. *p* values < 0.05 < 0.01 < 0.001 were considered statistically significant.

## 12. Results

### 12.1. Differential Expression Landscapes in PVTT and Hypertension Cohorts

**Figure 1:**
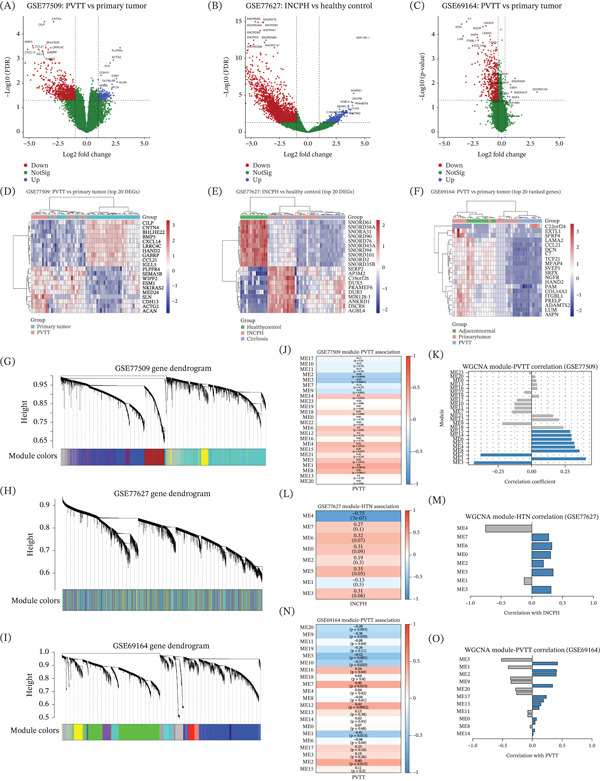
Differential expression signatures and WGCNA‐based identification of disease‐associated gene modules in PVTT and portal hypertension transcriptomic datasets. Volcano plots for differential expression comparisons: (A) GSE77509, PVTT versus primary tumor; (B) GSE77627, HTN versus healthy control; and (C) GSE69164, PVTT versus primary tumor. Points are colored by differential expression status (upregulated, downregulated, or not significant). (D–F) Heatmaps of the Top 20 differentially expressed genes for each dataset. Heatmaps show scaled expression values with hierarchical clustering of both genes and samples and group annotations above each heatmap. (G–I) Gene dendrograms and module color assignments generated by WGCNA for GSE77509, GSE77627, and GSE69164, respectively. Genes are hierarchically clustered based on topological overlap with modules identified using dynamic tree cutting. (J, L, N) Heatmaps showing correlations between module eigengenes and disease traits: PVTT in GSE77509 and GSE69164 and HTN in GSE77627. (K, M, O) Bar plots summarizing module–trait correlation coefficients for each dataset.

Volcano plots show the global distribution of effect sizes (log2 fold change) and statistical significance (−log10 FDR) for each comparison. Across all three datasets, a substantial number of genes met the predefined differential expression thresholds in their respective comparisons (Figure 1A–C). Figure 1D–F illustrates the Top 20 DEGs (ranked by differential expression statistics within each dataset) using hierarchical clustering heatmaps.

### 12.2. WGCNA Identifies PVTT‐ and HTN‐Associated Coexpression Modules

Gene dendrograms illustrate hierarchical clustering of genes based on topological overlap, with modules assigned by dynamic tree cutting in GSE77509 (Figure 1G), GSE77627 (Figure 1H), and GSE69164 (Figure 1I). Module–trait relationships were subsequently assessed by correlating module eigengenes with disease status. In GSE77509, several modules showed statistically significant correlations with PVTT status, as visualized in the module–PVTT association heatmap (Figure 1J). The corresponding bar plot summarizes the direction and magnitude of correlation coefficients for each module with PVTT (Figure 1K). In the hypertension dataset GSE77627, module–trait association analysis identified multiple modules correlated with HTN status (Figure 1L). The strength and direction of these associations are further illustrated in the module–HTN correlation bar plot (Figure 1M). Similarly, GSE69164 revealed a subset of modules significantly correlated with PVTT status (Figure 1N), with correlation coefficients summarized in the corresponding bar plot (Figure 1O).

### 12.3. Quantification of Bleeding Risk Proxy Biological Programs

**Figure 2:**
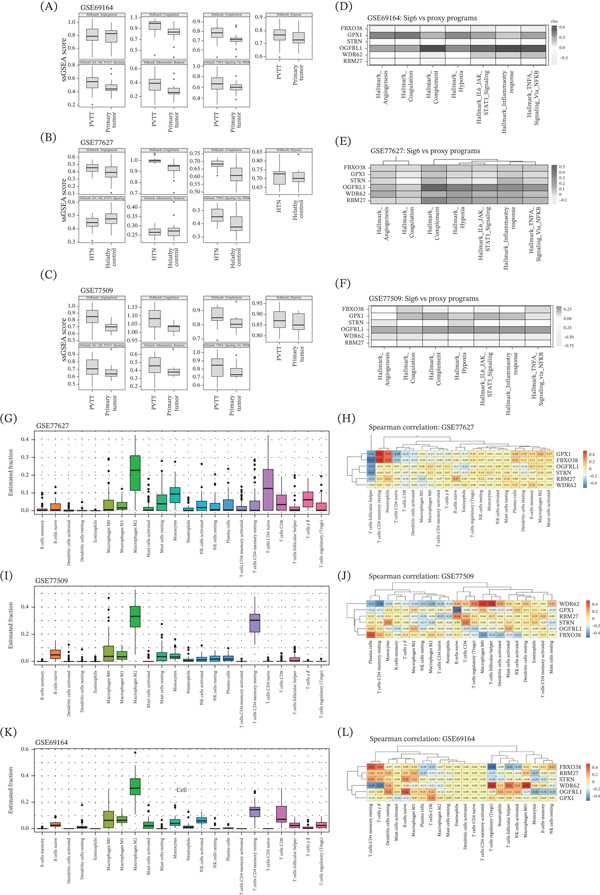
Bleeding risk proxy biological programs, immune infiltration profiling, and single‐cell RNA‐seq analysis of a six‐gene signature. (A–C) Boxplots showing ssGSEA scores for hallmark pathways related to angiogenesis, coagulation, complement activation, hypoxia, IL6–JAK–STAT3 signaling, inflammatory response, and TNF*α* signaling via NF‐*κ*B. (D–F) Heatmaps showing Spearman′s correlation coefficients between expression levels of the six‐gene signature and ssGSEA pathway scores in GSE69164, GSE77627, and GSE77509. Color intensity represents the magnitude and direction of correlation (*ρ*). Boxplots showing CIBERSORTx‐estimated immune cell fractions for 22 immune cell subsets in (G) GSE77627, (I) GSE77509, and (K) GSE69164. Heatmaps depicting Spearman′s correlation coefficients between expression levels of the six‐gene signature and estimated immune cell fractions in (H) GSE77627, (J) GSE77509, and (L) GSE69164. Color intensity represents the magnitude and direction of correlation (*ρ*).

To quantify molecular programs relevant to gastrointestinal bleeding risk, ssGSEA was applied to hallmark pathways associated with coagulation, complement activation, angiogenesis, hypoxia, inflammatory response, IL6–JAK–STAT3 signaling, and TNF*α* signaling via NF‐*κ*B. In all datasets, pathway activity scores for these biological programs were compared between PVTT and primary tumor samples (Figure 2A–C). Several hallmark pathways displayed differential ssGSEA score distributions between the two groups within each dataset, which indicate dataset‐specific alterations in pathway‐level activity associated with PVTT status. Spearman′s correlation analysis was performed between gene expression levels and ssGSEA pathway scores within each dataset. Heatmaps illustrate gene–pathway correlation patterns for GSE69164 (Figure 2D), GSE77627 (Figure 2E), and GSE77509 (Figure 2F). The strength and direction of correlations varied across genes, pathways, and datasets.

### 12.4. Immune Cell Infiltration and Gene–Immune Correlations

In the HTN dataset GSE77627, the distribution of estimated immune cell fractions across samples is shown in Figure 2G. The immune landscape comprised multiple myeloid and lymphoid populations with variable abundance across samples. In GSE77627, gene–immune correlations varied across genes and immune cell types, with both positive and negative correlation coefficients observed (Figure 2H). In GSE77509, immune cell fraction distributions are shown in Figure 2I, and gene–immune correlation patterns are summarized in Figure 2J. Distinct correlation structures were observed across genes and immune subsets, which reflect dataset‐specific associations between transcript levels and immune cell estimates. In GSE69164, immune infiltration profiles are presented in Figure 2K with corresponding Spearman correlation heatmaps shown in Figure 2L. Correlations differed by gene and immune cell type, without uniform trends across all datasets.

### 12.5. Single‐Cell Transcriptomic Landscape and Cell Type–Specific Expression of the Six‐Gene Signature in PVTT

**Figure 3:**
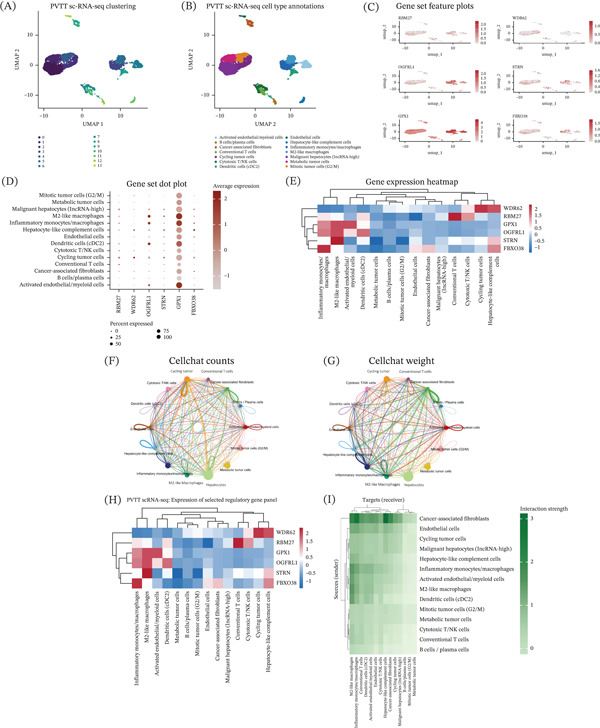
Single‐cell RNA‐seq analysis of six‐gene signature. (A) UMAP visualization of unsupervised clustering of PVTT single‐cell transcriptomes, showing distinct cellular clusters. (B) UMAP plot with annotated cell types based on canonical marker gene expression. (C) Feature plots showing the expression of RBM27, WDR62, OGFRL1, STRN, GPX1, and FBXO38 across the UMAP embedding. (D) Dot plot summarizing the percentage of expressing cells and average expression levels of the six genes across annotated cell types. (E) Heatmap of pseudobulk expression profiles for the six‐gene signature across cell types. (F) CellChat network showing the number of inferred ligand–receptor interactions between annotated cell types (interaction counts). (G) CellChat network weighted by interaction strength, illustrating relative communication probabilities between cell populations. (H) Heatmap showing pseudobulk expression of the six‐gene signature across annotated cell types. (I) Sender–receiver heatmap summarizing aggregated ligand–receptor interaction strengths between cell types, with rows representing signal senders and columns representing signal receivers.

Unsupervised graph‐based clustering identified multiple transcriptionally distinct cell populations, visualized using UMAP (Figure 3A). Clusters were subsequently annotated based on canonical marker gene expression, resulting in the identification of malignant hepatocytes, endothelial cells, fibroblasts, immune cell subsets, and other tumor‐associated cell populations (Figure 3B). The expression patterns of the six‐gene signature were visualized across the UMAP embedding using feature plots (Figure 3C). These plots demonstrate heterogeneous expression of individual genes across distinct cellular clusters. To further quantify the distribution of the six genes across annotated cell types, dot plot analysis was performed (Figure 3D). This representation summarizes both the proportion of cells expressing each gene and the average expression level within each cell type. Pseudobulk expression profiles were generated, followed by hierarchical clustering and visualization as a heatmap (Figure 3E). This analysis further illustrates differential expression patterns of the six‐gene signature across cell types, and clustering reflects similarities and differences in gene expression profiles among tumor, stromal, endothelial, and immune populations.

### 12.6. Cell–Cell Communication Networks in PVTT Single‐Cell Transcriptomic Data

Global interaction networks summarizing the number of inferred ligand–receptor interactions between annotated cell types are shown in Figure 3F. The network illustrates extensive connectivity among tumor, stromal, endothelial, and immune cell populations with interactions distributed across multiple sender–receiver pairs. Interaction strength–weighted networks are shown in Figure 3G, where edge thickness reflects aggregated communication probability between cell types. The weighted representation highlights variation in the relative strength of signaling between different cellular compartments. To contextualize cell–cell communication within the transcriptional landscape, expression of the six‐gene signature was visualized across annotated cell types using a pseudobulk heatmap (Figure 3H). Aggregated ligand–receptor interaction strengths were summarized in a sender–receiver heatmap (Figure 3I) where rows represent signal‐sending cell types and columns represent signal‐receiving cell types.

### 12.7. Expression Profiling of the Six‐Gene Signature Across LIHC Tumor Status and Pathological Stages

**Figure 4:**
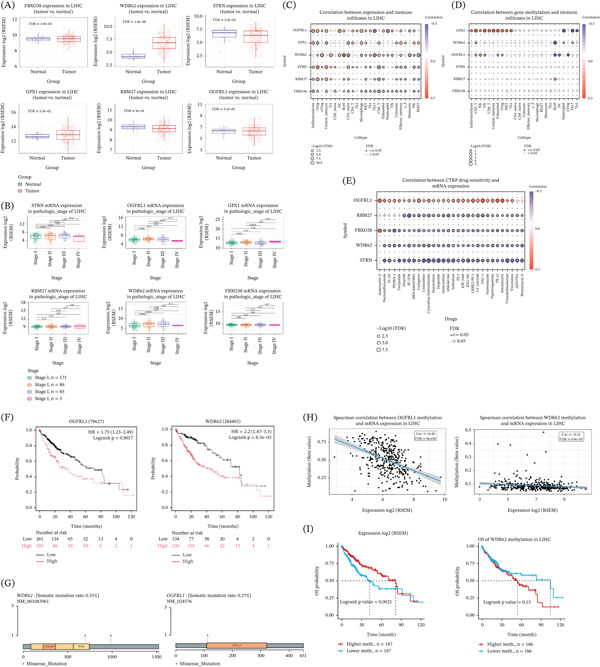
Expression patterns and correlation analyses of the six‐gene signature in LIHC. (A) Boxplots showing mRNA expression levels of FBXO38, WDR62, STRN, GPX1, RBM27, and OGFRL1 in LIHC tumor versus normal liver tissues. Expression values are shown as log2 (RSEM), with false discovery rate values indicated for each comparison. (B) Boxplots showing mRNA expression of the six genes across pathological stages (Stages I–IV) of LIHC. (C) Bubble plot showing Spearman′s correlations between mRNA expression of the six‐gene signature and immune cell infiltration levels in LIHC. Color represents correlation direction and magnitude, and bubble size corresponds to −log10 (FDR). (D) Bubble plot showing correlations between DNA methylation levels of the six genes and immune cell infiltration in LIHC, visualized using the same statistical encoding. (E) Bubble plot illustrating correlations between gene mRNA expression and CTRP drug sensitivity scores across multiple compounds. (F) Kaplan–Meier overall survival curves stratified by high and low mRNA expression of OGFRL1 (left) and WDR62 (right) in LIHC, with hazard ratios, 95% confidence intervals, and log‐rank *p* values indicated. (G) Schematic representations of somatic mutation profiles for WDR62 and OGFRL1, showing mutation frequency and genomic distribution. (H) Scatter plots showing Spearman′s correlations between DNA methylation levels and mRNA expression for OGFRL1 (left) and WDR62 (right) in LIHC, with correlation coefficients and FDR values reported. (I) Kaplan–Meier overall survival curves stratified by high and low DNA methylation levels of OGFRL1 (left) and WDR62 (right), with log‐rank *p* values indicated.

Boxplots illustrate the distribution of mRNA expression for shortlisted genes across tumor and normal groups, with FDR values reported for each comparison (Figure 4A). These comparisons demonstrate gene‐specific differences in expression distributions between tumor and normal samples under the applied statistical framework. To further characterize expression dynamics across disease progression, mRNA expression levels of the six genes were examined across pathological stages of LIHC. Stage‐stratified boxplots display expression distributions for Stages I, II, III, and IV, with pairwise statistical comparisons annotated where applicable (Figure 4B). Expression levels varied across stages in a gene‐dependent manner, with differences observed between early and advanced pathological stages for selected genes.

### 12.8. Associations Between Gene Expression, DNA Methylation, Immune Infiltrates, and Drug Sensitivity in LIHC

Bubble plots display Spearman′s correlation coefficients between gene expression levels and immune cell types, with color indicating correlation direction and magnitude and point size reflecting statistical significance (Figure 4C). Across genes and immune subsets, both positive and negative correlations were observed with variability in statistical significance across cell types. The corresponding bubble plot summarizes Spearman′s correlations between methylation levels of each gene and immune cell fractions (Figure 4D). Correlation patterns differed from those observed at the mRNA level and varied across genes and immune cell types, indicating that methylation–immune associations are gene‐ and cell type–specific within the analyzed cohort. The bubble plot illustrates correlations between the expression of each gene and sensitivity scores across multiple compounds, with correlation direction, magnitude, and statistical significance encoded visually (Figure 4E).

### 12.9. Survival Associations, Somatic Mutation Profiles, and Methylation–Expression Relationships of OGFRL1 and WDR62 in LIHC

Overall survival analysis stratified by gene expression levels demonstrated a significant association between higher expression of OGFRL1 and reduced overall survival in LIHC (hazard ratio 1.75, 95% CI 1.23–2.49; log‐rank *p* = 0.0017). Similarly, elevated WDR62 expression was associated with poorer overall survival (hazard ratio 2.2, 95% CI 1.47–3.3; log‐rank *p* = 8.5 × 10^−5^; Figure 4F). Somatic mutation analysis revealed low mutation frequencies for both genes in LIHC. WDR62 exhibited a somatic mutation rate of 0.55%, while OGFRL1 showed a mutation rate of 0.27% across analyzed samples (Figure 4G). To evaluate epigenetic associations, correlations between DNA methylation levels and mRNA expression were assessed. A moderate negative correlation was observed between OGFRL1 methylation and its mRNA expression (Spearman′s *r* = −0.48, FDR = 0). WDR62 methylation also showed a negative correlation with mRNA expression, although with a smaller effect size (Spearman′s *r* = −0.21, FDR = 4.9 × 10^−5^) (Figure 4H). Overall survival was additionally evaluated based on methylation status. For OGFRL1, higher methylation levels were associated with differential overall survival compared to lower methylation levels, with a significant log‐rank *p* value of 0.0021. In contrast, methylation‐based survival stratification for WDR62 did not reach statistical significance (log‐rank *p* = 0.15) (Figure 4I).

### 12.10. PPI Networks and Gene Ontology Enrichment of OGFRL1 and WDR62

**Figure 5:**
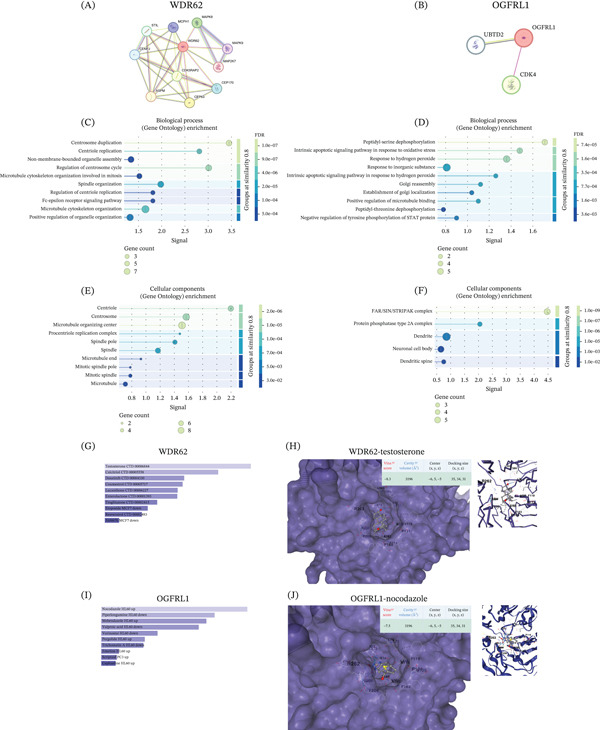
Protein–protein interaction networks, Gene Ontology enrichment, and docking analysis of OGFRL1 and WDR62. (A) STRING‐derived protein–protein interaction network for WDR62. (B) STRING‐derived protein–protein interaction network for OGFRL1. (C, D) Gene Ontology biological process enrichment for genes associated with WDR62 and OGFRL1. (E, F) Gene Ontology cellular component enrichment for genes associated with WDR62 and OGFRL1. (G) Drug signature enrichment results for WDR62, highlighting testosterone as the top‐ranked compound. (H) Drug signature enrichment results for OGFRL1, highlighting nocodazole as the top‐ranked compound. (I) Molecular docking of testosterone with WDR62, showing the predicted binding cavity, ligand pose, cavity volume (3196 Å^3^), docking center (−6.5, 5.0, and −5.0), docking size (35, 34, and 31 Å), and Vina score (−8.3 kcal/mol). (J) Molecular docking of nocodazole with OGFRL1, showing the predicted binding cavity, ligand pose, cavity volume (3196 Å^3^), docking center (−6.5, 5.0, and −5.0), docking size (35.34 Å), and Vina score (−7.5 kcal/mol).

PPI networks were constructed for WDR62 and OGFRL1 using the STRING database to summarize known and predicted interaction partners. The WDR62 interaction network consisted of multiple interacting nodes forming a densely connected subnetwork, whereas the OGFRL1 network contained a smaller number of interacting partners with fewer connections (Figure 5A,B). For WDR62, biological process enrichment highlighted terms related to centrosome duplication, spindle organization, microtubule‐based processes, and regulation of cell cycle–associated structures (Figure 5C). Cellular component enrichment for WDR62‐associated genes included centrosome, centriolar satellite, spindle, and microtubule‐related components (Figure 5E). For OGFRL1, biological process enrichment identified terms associated with peptide dephosphorylation, intracellular signaling pathways, response to growth factors, and regulation of phosphorylation‐related processes (Figure 5D). Cellular component enrichment analysis for OGFRL1‐associated genes highlighted annotations including protein phosphatase complexes, dendrites, neuronal cell bodies, and dendritic spines (Figure 5F).

### 12.11. Drug Signature Prioritization and Molecular Docking of WDR62 and OGFRL1

Drug signature analysis identified candidate compounds associated with WDR62 and OGFRL1 expression profiles. For WDR62, testosterone emerged as the top‐ranked compound among enriched drug signatures (Figure 5G). For OGFRL1, nocodazole was identified as the leading candidate compound based on enrichment ranking (Figure 5H). Molecular docking was subsequently performed to evaluate the structural compatibility between WDR62 and testosterone and between OGFRL1 and nocodazole. For the WDR62‐testosterone complex, CB‐Dock2 identified a binding cavity with a cavity volume of 3196 Å^3^. The predicted docking center coordinates were −6.5, 5, and −5.0 (*x*, *y*, *z*), with a docking box size of 35, 34, and 31 Å. The predicted binding affinity (Vina score) for testosterone binding to WDR62 was −8.3 kcal/mol (Figure 5I). The docking pose shows testosterone positioned within the predicted binding pocket of WDR62, with surrounding residues displayed for spatial reference. For the OGFRL1–nocodazole complex, docking analysis similarly identified a cavity volume of 3196 Å^3^. The predicted docking center coordinates were −6.5, 5.0, and −5.0 (*x*, *y*, *z*), with a docking box size of 35, 34, and 31 Å. The predicted Vina binding score for nocodazole binding to OGFRL1 was −7.5 kcal/mol (Figure 5J). The docking pose illustrates nocodazole localized within the predicted binding cavity of OGFRL1, with nearby residues visualized to depict the interaction environment.

### 12.12. OGFRL1 and WDR62 Knockdowns Suppress HCC Cell Growth and Motility In Vitro

**Figure 6:**
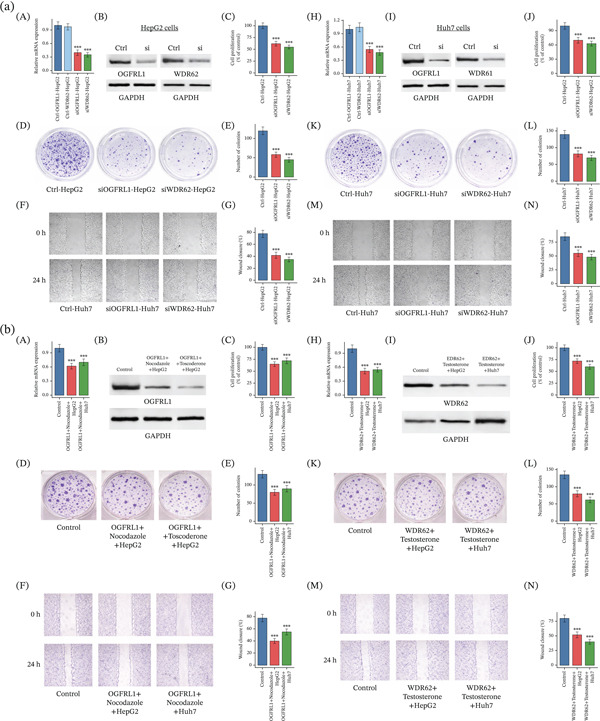
OGFRL1 and WDR62 knockdown suppress proliferation, clonogenicity, and migration in HepG2 and Huh7 cells. (a): (A) RT–qPCR analysis showing efficient siRNA‐mediated knockdown of OGFRL1 and WDR62 mRNA expression in HepG2 cells relative to control. (B) Western blot analysis confirming reduced protein expression of OGFRL1 and WDR62. (C) Cell proliferation assay demonstrating significantly reduced proliferative capacity in siOGFRL1‐ and siWDR62‐treated HepG2 cells compared with control. (D) Representative images of colony formation assays showing reduced clonogenic growth following OGFRL1 or WDR62 knockdown. (E) Quantification of colony numbers corresponding to (D). (F) Representative wound healing assay images captured at 0 and 24 h postscratch. (G) Quantification of wound closure percentage demonstrating impaired migratory capacity in knockdown cells. (H) RT–qPCR analysis showing significant downregulation of OGFRL1 and WDR62 transcripts in Huh7 cells following siRNA transfection. (I) Western blot validation of OGFRL1 and WDR62 protein knockdown with GAPDH as a loading control. (J) Cell proliferation assay indicating reduced growth of siOGFRL1‐ and siWDR62‐treated Huh7 cells compared with control. (K) Representative colony formation assay images demonstrating decreased colony density upon gene knockdown. (L) Quantitative analysis of colony numbers corresponding to (D). (M) Representative wound healing assay images at 0 and 24 h after scratch generation. (N) Quantification of wound closure percentage showing significantly impaired migration in OGFRL1‐ and WDR62‐silenced cells. (b) Nocodazole treatment phenocopies OGFRL1 and WDR62 knockdown and suppresses malignant behaviors in HCC cells. (A) RT–qPCR analysis showing reduced OGFRL1 mRNA expression in HepG2 and Huh7 cells treated with nocodazole compared with control. (B) Western blot analysis confirming decreased OGFRL1 protein levels following nocodazole treatment, with GAPDH as a loading control. (C) Cell proliferation assay demonstrating significantly reduced proliferation following nocodazole exposure. (D) Representative colony formation assay images illustrating decreased clonogenic growth in nocodazole‐treated cells. (E) Quantification of colony numbers corresponding to (D). (F) Representative wound healing assay images at 0 and 24 h postscratch showing delayed wound closure after nocodazole treatment. (G) Quantitative analysis of wound closure percentage indicating impaired migratory capacity. (H) RT–qPCR analysis demonstrating significant downregulation of WDR62 mRNA expression in HepG2 and Huh7 cells treated with testosterone. (I) Western blot validation of reduced WDR62 protein levels following testosterone treatment, with GAPDH serving as a loading control. (J) Cell proliferation assay showing reduced growth rates in testosterone‐treated cells compared with controls. (K) Representative images of colony formation assays illustrating diminished clonogenic capacity following testosterone exposure. (L) Quantification of colony numbers corresponding to (D). (M) Representative wound healing assay images at 0 and 24 h demonstrating delayed wound closure after testosterone treatment. (N) Quantification of wound closure percentage confirming impaired migratory behavior in treated cells.

Silencing of OGFRL1 and WDR62 in HCC cells resulted in consistent suppression of malignant phenotypes across two independent models. In HepG2 cells, siRNA‐mediated knockdown significantly reduced mRNA expression of both genes (Figure 6a, A; *p* < 0.001), which was confirmed at the protein level by Western blotting with stable GAPDH loading (Figure 6a, B and Supporting Information 1). Functional assays demonstrated that depletion of either gene markedly inhibited cell proliferation (Figure 6a, C; *p* < 0.001), reduced clonogenic capacity as evidenced by fewer and smaller colonies (Figure 6a, D,E; *p* < 0.001), and impaired wound closure in scratch assays, indicating reduced migratory ability (Figure 6a, F,G; *p* < 0.001). Similar effects were observed in Huh7 cells, where OGFRL1 and WDR62 knockdown significantly decreased transcript and protein levels (Figure 6a, H,I, Supporting Information 1; *p* < 0.001), suppressed proliferation (Figure 6a, J; *p* < 0.001), diminished colony formation (Figure 6a, K,L; *p* < 0.001), and significantly delayed wound closure compared with controls (Figure 6a, M,N; *p* < 0.001).

### 12.13. Drug Treatment Phenocopy Functional Effects of OGFRL1 and WDR62 Perturbation

Pharmacological treatment of HCC cells with compounds predicted against OGFRL1 and WDR62 recapitulated the growth‐ and migration‐inhibitory phenotypes observed following genetic knockdown. In Figure 6, treatment with nocodazole, the compound prioritized for OGFRL1, significantly reduced OGFRL1 mRNA expression in both HepG2 and Huh7 cells compared with control (Figure 6b, A; *p* < 0.001), and this reduction was confirmed at the protein level by Western blotting (Figure 6b, B and Supporting Information 1). Functionally, nocodazole‐treated cells exhibited markedly decreased proliferative capacity (Figure 6b, C; *p* < 0.001), reduced clonogenic survival as demonstrated by fewer colonies (Figure 6b, D,E; *p* < 0.001), and impaired wound closure in scratch assays (Figure 6b, F,G; *p* < 0.001), indicating suppressed migratory behavior. Similarly, treatment with testosterone, identified through docking as a candidate modulator of WDR62, significantly downregulated WDR62 expression at both the mRNA (Figure 6b, H; *p* < 0.001) and protein levels (Figure 6b, I and Supporting Information 1). Testosterone exposure led to a pronounced reduction in cell proliferation (Figure 6b, J; *p* < 0.001), diminished colony‐forming ability (Figure 6b, K,L; *p* < 0.001), and significantly delayed wound closure in both HepG2 and Huh7 cells (Figure 6b, M,N; *p* < 0.001).

## 13. Discussion

PVTT represents a critical clinical and biological inflection point in HCC, where malignant progression intersects with profound vascular remodeling and portal hypertension–related complications [39]. Despite its clinical significance, the molecular determinants linking PVTT, portal hypertension, immune dysregulation, and gastrointestinal bleeding risk remain poorly characterized [40]. In this study, we applied a multilayered integrative framework combining bulk transcriptomics, coexpression network analysis, pathway‐level quantification, immune deconvolution, single‐cell resolution, epigenetic profiling, and structural drug interrogation to delineate shared and disease‐relevant molecular programs across PVTT and noncirrhotic portal hypertension.

Differential expression analyses across three independent datasets demonstrated robust transcriptomic alterations associated with PVTT and portal hypertension–related conditions, providing a reproducible foundation for downstream integration [41]. Rather than relying on single‐cohort findings, we leveraged WGCNA to identify coexpression modules associated with disease status within each dataset, thereby capturing coordinated transcriptional programs that may be obscured by gene‐centric approaches [42]. Intersecting module‐derived hub genes with differentially expressed genes across all datasets yielded a concise six‐gene signature representing genes consistently implicated across PVTT and portal hypertension contexts. To contextualize these genes within clinically relevant vascular complications, we quantified pathway‐level activity of hallmark biological programs associated with gastrointestinal bleeding risk, including coagulation, complement activation, inflammatory signaling, hypoxia, angiogenesis, and cytokine‐mediated pathways [43]. ssGSEA revealed dataset‐specific but consistent alterations in these programs between PVTT or portal hypertension samples and their respective controls. Correlation analyses further demonstrated heterogeneous associations between individual signature genes and these pathway scores, underscoring that bleeding risk–associated biology is distributed across multiple molecular axes rather than driven by a single pathway [44].

Immune infiltration analysis provided additional resolution by characterizing the immune landscapes associated with these transcriptional changes [45]. Across datasets, estimated immune cell fractions exhibited substantial variability, reflecting heterogeneous immune microenvironments in PVTT and portal hypertension. Gene–immune correlation analyses revealed both positive and negative associations that differed by gene and immune subset, indicating that the six‐gene signature does not uniformly track with a single immune phenotype [46]. Importantly, these results were interpreted descriptively, as computational deconvolution reflects relative immune abundance rather than direct functional activity. Single‐cell RNA sequencing analysis further refined the spatial and cellular context of the six‐gene signature within PVTT tissue. The genes exhibited heterogeneous, cell type–specific expression patterns across malignant hepatocytes, stromal cells, endothelial compartments, and immune populations, rather than uniform expression across all cells. Pseudobulk and dot plot analyses reinforced this heterogeneity, highlighting that the signature reflects contributions from multiple cellular compartments [47]. Cell–cell communication analysis demonstrated extensive intercellular signaling among tumor, immune, stromal, and endothelial cells, providing a structural framework within which these genes may operate, without attributing functional dominance or directional signaling roles [48].

Translational relevance was explored through GSCA‐based analyses in LIHC, where expression profiling across tumor versus normal tissue and pathological stages revealed gene‐specific expression differences. Integration of immune correlations, DNA methylation, and drug sensitivity analyses demonstrated that transcriptional regulation of these genes is shaped by multiple regulatory layers [49]. Survival analyses prioritized OGFRL1 and WDR62 as the only genes within the six‐gene signature with statistically significant associations with overall survival at an FDR below 5%. Importantly, somatic mutation frequencies for both genes were low, suggesting that transcriptional and epigenetic mechanisms, rather than recurrent coding mutations, may underlie their dysregulation. Epigenetic analyses supported this notion, as both OGFRL1 and WDR62 exhibited inverse correlations between DNA methylation and mRNA expression, with methylation‐based survival stratification reaching significance for OGFRL1 [50]. These findings highlight the relevance of epigenetic regulation in shaping prognostic gene expression patterns in LIHC. Furthermore, correlation analyses with fibrosis‐ and tumor‐associated genes, including ITGA6, COL11A, and TGFB1, revealed coordinated expression patterns, positioning OGFRL1 and WDR62 within broader transcriptional programs linked to extracellular matrix remodeling and tumor progression, without implying direct regulatory interactions. Structural and drug signature analyses extended these findings toward therapeutic hypothesis generation. Drug signature enrichment identified testosterone and nocodazole as candidate compounds associated with WDR62 and OGFRL1, respectively [51]. Molecular docking analyses demonstrated structurally feasible binding within predicted cavities, with favorable Vina scores under standardized docking parameters [52]. Docking results were interpreted qualitatively based on predicted binding affinity and pose consistency, and RMSD benchmarking against reference ligands was performed, which represents a limitation of the structural analysis and should be covered in future analyses.

The functional experiments presented here provide important biological validation of the computationally identified immune–coagulation signature and establish OGFRL1 and WDR62 as key regulators of aggressive HCC phenotypes. Consistent with their association with poor overall survival and epigenetically regulated expression in patient cohorts, genetic silencing of either gene markedly suppressed proliferation, clonogenic growth, and migratory capacity in two independent HCC cell lines, supporting a direct role in tumor progression rather than a context‐dependent or cell line–specific effect. Notably, pharmacological targeting with nocodazole and testosterone, compounds prioritized through integrative drug signature analysis and molecular docking, phenocopied the effects of gene knockdown, further strengthening the functional relevance of these targets and linking transcriptional dysregulation to actionable molecular vulnerabilities. Although nocodazole and testosterone have pleiotropic cellular effects, their consistent suppression of OGFRL1‐ and WDR62‐associated phenotypes aligns with the predicted protein–ligand interactions and suggests that these genes participate in pathways governing cytoskeletal dynamics, cell cycle progression, and migratory behavior. Importantly, these tumor‐intrinsic effects integrate conceptually with our transcriptomic and single‐cell analyses showing immune–coagulation and vascular remodeling programs in PVTT and portal hypertension, supporting a model in which dysregulated tumor cell behavior, immune signaling, and vascular instability converge to increase bleeding risk in advanced HCC.

## 14. Conclusion

This study provides an integrated multiomics framework linking PVTT and portal hypertension to gastrointestinal bleeding risk in HCC. By combining differential expression analysis, coexpression network modeling, pathway‐level enrichment, immune deconvolution, and single‐cell resolution, we identified a robust six‐gene signature that consistently associates with vascular and inflammatory disruptions across independent datasets. The enrichment of biological programs such as coagulation, complement activation, angiogenesis, hypoxia, and inflammatory signaling underscores the relevance of this signature to bleeding‐prone portal hypertensive conditions. Single‐cell transcriptomic analysis further revealed that the expression of these genes is not confined to malignant hepatocytes but is also present in endothelial, stromal, and immune cells, highlighting the multicellular contributions to the bleeding‐associated pathophysiology. Moreover, cell–cell communication analysis demonstrated extensive intercellular signaling, suggesting coordinated immune–vascular remodeling as a key driver of disease progression. Among the six identified genes, OGFRL1 and WDR62 emerged as highly prognostic, with significant associations to overall survival and epigenetic regulation through DNA methylation, linking these genes to both tumor biology and vascular complications. Additionally, functional validation of these genes through knockdown and drug treatments further affirmed their role in regulating HCC cell proliferation, migration, and clonogenic survival. These findings not only enhance our understanding of the molecular mechanisms driving bleeding risk in advanced HCC but also position OGFRL1 and WDR62 as promising therapeutic targets for managing portal hypertension and related complications in HCC patients.

## Funding

No funding was received for this manuscript.

## Ethics Statement

The authors have nothing to report.

## Consent

The authors have nothing to report.

## Conflicts of Interest

The authors declare no conflicts of interest.

## Supporting information


**Supporting Information** Additional supporting information can be found online in the Supporting Information section. Uncropped Western blot bands.

## Data Availability

The data that support the findings of this study are available from the corresponding authors upon reasonable request.
