# Supplementary figures and images for: Convergent Immune–Coagulation Programs Underlie Gastrointestinal Bleeding Risk in Portal Vein Tumor Thrombosis–Associated Hepatocellular Carcinoma and Portal Hypertension
Source: Hum Mutat. 2026 May 12;2026:7210691. doi: 10.1155/humu/7210691 (PMC13162123; doi:10.1155/humu/7210691)

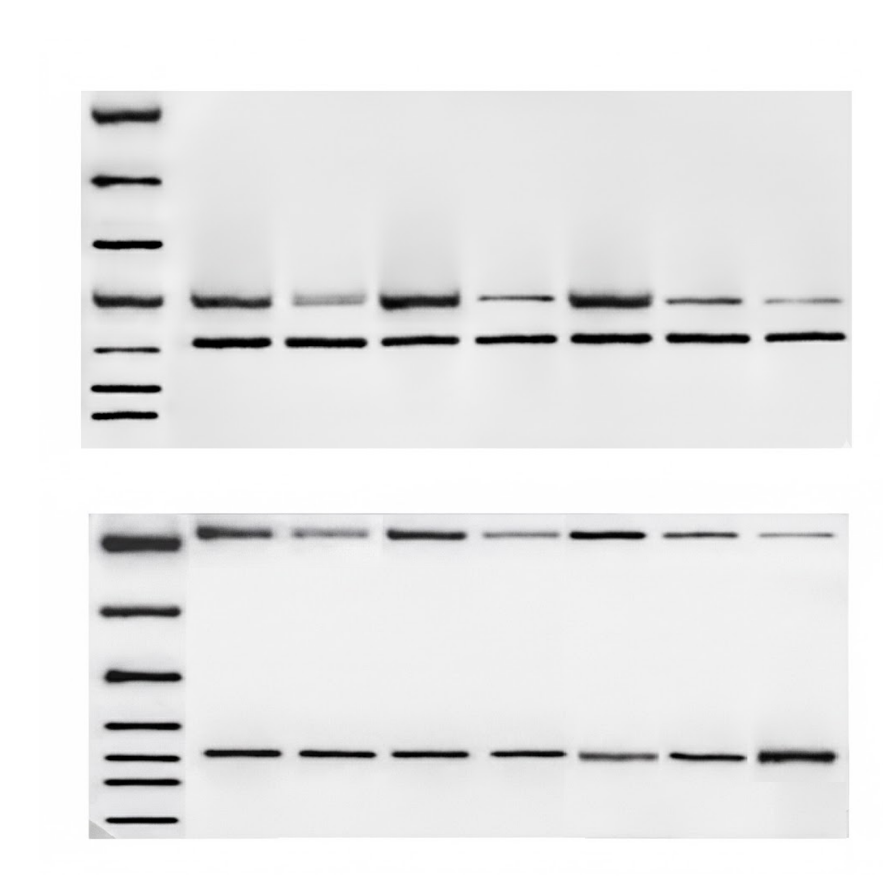


**Uncropped Western blot bands**

Supplement: Supplementary file 1 — Supporting Information Additional supporting information can be found online in the Supporting Information section. Uncropped Western blot bands. [file HUMU-2026-7210691-s001.docx]
